# The British antibiotic and silver-impregnated catheters for ventriculoperitoneal shunts multi-centre randomised controlled trial (the BASICS trial): study protocol

**DOI:** 10.1186/1745-6215-15-4

**Published:** 2014-01-03

**Authors:** Michael D Jenkinson, Carrol Gamble, John C Hartley, Helen Hickey, Dyfrig Hughes, Michaela Blundell, Michael J Griffiths, Tom Solomon, Conor L Mallucci

**Affiliations:** 1 Department of Neurosurgery, The Walton Centre NHS Foundation Trust, Lower Lane, Fazakerley, Liverpool L9 7LJ, UK; 2 Institute of Infection and Global Health, University of Liverpool, The Ronald Ross Building, 8 West Derby Street, Liverpool L69 7BE, UK; 3 Medicines for Children Research Network (MCRN) Clinical Trials Unit, University of Liverpool Division of Child Health, Institute of Child Health, Royal Liverpool Children’s Hospital, Alder Hey, Liverpool L12 2AP, UK; 4 Department of Microbiology, Great Ormond Street Children’s Hospital, Great Ormond Street, London WC1N 3JH, UK; 5 Centre for Health Economics and Medicines Evaluation, Bangor University, Bangor, Gwynedd LL57 1UT, UK; 6 Department of Paediatric Neurosurgery, Alder Hey Children’s Hospital NHS Foundation Trust, Eaton Road, West Derby, Liverpool L12 2AP, UK; 7 Department of Neurology, The Walton Centre NHS Foundation Trust, Lower Lane, Fazakerley, Liverpool L9 7LJ, UK; 8 Department of Paediatric Neurology, Alder Hey Children’s Hospital NHS Foundation Trust, Eaton Road, Liverpool L12 2AP, UK

**Keywords:** Ventriculoperitoneal shunt, Infection, Clinical trial, Antibiotic-impregnated, Silver-impregnated

## Abstract

**Background:**

Insertion of a ventriculoperitoneal shunt (VPS) for the treatment of hydrocephalus is one of the most common neurosurgical procedures in the UK, but failures caused by infection occur in approximately 8% of primary cases. VPS infection is associated with considerable morbidity and mortality and its management results in substantial cost to the health service. Antibiotic-impregnated (rifampicin and clindamycin) and silver-impregnated VPS have been developed to reduce infection rates. Whilst there is some evidence showing that such devices may lead to a reduction in VPS infection, there are no randomised controlled trials (RCTs) to support their routine use.

**Methods/design:**

Overall, 1,200 patients will be recruited from 17 regional neurosurgical units in the UK and Ireland. Patients of any age undergoing insertion of their first VPS are eligible. Patients with previous indwelling VPS, active and on-going cerebrospinal fluid (CSF) or peritoneal infection, multiloculated hydrocephalus requiring multiple VPS or neuroendoscopy, and ventriculoatrial or ventriculopleural shunt planned will be excluded. Patients will be randomised 1:1:1 to either standard silicone (comparator), antibiotic-impregnated, or silver-impregnated VPS. The primary outcome measure is time to VPS infection. Secondary outcome measures include time to VPS failure of any cause, reason for VPS failure (infection, mechanical failure, or patient failure), types of bacterial VPS infection (organism type and antibiotic resistance), and incremental cost per VPS failure averted.

**Discussion:**

The British antibiotic and silver-impregnated catheters for ventriculoperitoneal shunts multi-centre randomised controlled trial (the BASICS trial) is the first multi-centre RCT designed to determine whether antibiotic or silver-impregnated VPS reduce early shunt infection compared to standard silicone VPS. The results of this study will be used to inform current neurosurgical practice and may potentially benefit patients undergoing shunt surgery in the future.

**Trial registration:**

International Standard Randomised Controlled Trial Number: ISRCTN49474281.

## Background

Hydrocephalus affects one in every 500 births, and is thus one of the most common causes of developmental disabilities in children. The condition also affects older children and adults of all ages and can be secondary to a variety of causes including intracranial tumours, haemorrhage, and infection. In the late 1950s, the development of treatment with cerebral shunts revolutionised the management of these patients. Standard treatment for hydrocephalus remains the ventriculoperitoneal shunt (VPS), which is composed of silicone tubing and a valve and which drains cerebrospinal fluid (CSF) from the ventricles into the peritoneal cavity. Insertion of a VPS for hydrocephalus is now one of the most common procedures performed in neurosurgical units, and between 3,000 and 3,500 shunt operations are carried out in adults and children per year in the UK [1]. Shunt failure due to infection has plagued this neurosurgical advance ever since it was developed. The incidence of shunt infection varies markedly in the literature from 3 to 27% [2-6] and is higher in certain groups, for example neonates, children under 1 year old, and patients treated with a previous temporary external ventricular drain (EVD). Episodes of shunt infection have a significant impact on patients, in terms of health-related quality of life, cognitive function and IQ [7], and survival, with the number of shunt infections being an independent predictor of death in patients requiring CSF shunts (HR 1.66, 95% CI: 1.02 to 2.72) [8]. Impacts on health services are also significant; these include the requirement for prolonged inpatient hospitalisation, additional surgery to remove the infected hardware, placement of a temporary EVD (a temporary tube placed in the ventricles that are prone to infection), intravenous and intrathecal antibiotics, and further surgery to place a new shunt once the infection has been treated.

Currently there are three types of shunt catheter available: standard, antibiotic-impregnated, and silver-impregnated. There is no evidence on their comparative effectiveness, and consequently practice is variable across the UK and worldwide, with selection being according to surgeon preference. The three main types of VPS catheters on the market are:

1. Standard VPS made of silicone.

2. Antibiotic-impregnated VPS made of silicone and impregnated with antibiotics (0.15% clindamycin and 0.054% rifampicin). This type has been on the market for approximately 9 years and has been adopted by the neurosurgical community as a means to potentially reduce shunt infection, despite the fact that it is more expensive and evidence is lacking. A recently published systematic review and meta-analysis of antibiotic-impregnated VPS identified one randomised controlled trial (RCT) and 11 observational studies. The RCT, conducted in a single centre in South Africa, demonstrated a trend favouring the antibiotic-impregnated VPS compared to standard VPS, but did not show a statistically significant difference between the two groups in the risk of shunt infection (RR: 0.38, CI: 0.11, 1.30; *P* = 0.12); however, a meta-analysis of the 11 observational studies showed a statistically significant difference favouring the antibiotic-impregnated VPS (RR: 0.37, CI: 0.23,0.60; *P* <0.0001) [9]. Research on antibiotic-impregnated VPS in Liverpool has shown that over a 2-year period the infection rate was reduced in paediatric patients compared to historical controls [10]. However continued data collection over 3.5 years and published as part of a Liverpool-led multi-centre observational study with two other UK paediatric neurosurgical units showed no significant reduction in infection in Liverpool [11]. Indeed the reduction in infection achieved by antibiotic-impregnated VPS in the multi-centre observational study was only seen in neonates and was heavily weighted by the results from one unit. This latter study [11] was not part of the published systematic review [9].

3. Silver-impregnated VPS made of silicone and impregnated with silver. This type was launched in the UK in March 2011 with similar infection claims. There is little doubt that silver ions have antimicrobial effects and they elute from silver-impregnated catheters. However the efficacy of silver-impregnated catheters at preventing VPS infections is not yet proven. There is one observational study of silver-impregnated VPS used to successfully treat seven patients with active CSF infection [12]. There are no observational studies comparing the silver-impregnated VPS infection rate with either the standard or antibiotic-impregnated VPS. However, in a RCT of EVDs in children and adults, silver-impregnated EVDs have been shown to reduce infection from 21.4% (30/140) to 12.3% (17/138) (*P* = 0.042) [13]. Two further observational studies comparing standard to silver-impregnated EVDs have also shown a reduction in infection rates [14,15]. The commentary accompanying the EVD study acknowledges the need for a RCT to study the relative benefits of silver-impregnated catheters.

Data from the UK shunt registry (to which most neurosurgical units contribute) show that 15% of shunt revisions are for infection [16]. In the largest randomised controlled shunt trial worldwide, the infection rate was 8.4% for primary VPS [17]. The commonest pathogens detected are *Staphylococcus* species, but in a proportion of patients with suspected infection the organism is never determined, especially if they have already received antimicrobial treatment or the organism is very slow growing, hampering microbiologically directed treatment selection. However newer molecular approaches are increasing the diagnostic rate for a range of central nervous system (CNS) infections. Whilst a systematic review [9] suggests that antibiotic-impregnated shunts may be an effective way of reducing the incidence of shunt infections, there is some evidence that antibiotic-impregnated VPS that become infected may be more difficult to treat leading to prolonged hospital stays [18]. Antibiotic-impregnated VPS prevent the sensitive organisms causing infection and may allow the rifampicin-resistant organisms to survive. Subsequently this may contribute to VPS failure secondary to infection. A second follow-up report of 500 antibiotic-impregnated primary and revision VPS insertions showed sensitive organisms may still cause infection but presentation was significantly delayed compared to standard historical controls [19]. It is plausible to suggest that antibiotic-impregnated VPS may lead to a delay in presentation allowing increased time for biofilm development on the ventricular surface rather than just the VPS. This could result in more difficult to treat, serious infection through rifampicin resistance, and warrants further investigation.

There are no economic evaluations of VPS, however four retrospective cost studies from the USA [20-23], which include both adults and children, suggest that cost savings may be possible through reduction of infection with antibiotic-impregnated shunts. Approximately 70% of shunt operations in the UK are with antibiotic-impregnated shunts (which cost £359) and there is likely to be a significant uptake of silver-impregnated (£365) shunts by neurosurgeons despite the lack of evidence of clinical benefit. Both VPS catheters cost more than standard (£137). The potential beneficial effect on health status of these impregnated catheters is reduced shunt infection and its negative sequelae. This must be balanced against the possibility of selecting out resistant organisms or rendering infections more difficult to treat with adverse impact on outcome and increased cost for impregnated VPS. The British antibiotic and silver impregnated catheters for ventriculoperitoneal shunts multi-centre randomised controlled trial (the BASICS trial) was therefore funded to determine whether these impregnated VPS reduce early infection, compared with standard VPS, and whether this is cost-effective.

### Primary objective

To determine whether antibiotic or silver-impregnated VPS reduce infection compared to standard VPS following insertion of the first *de novo* VPS.

### Secondary objectives

• To determine the proportion of first VPS infections occurring >6 months after insertion of *de novo* VPS.

• To determine whether antibiotic or silver-impregnated VPS reduce shunt failure of any cause compared to standard VPS following insertion of *de novo* VPS.

• To assess whether the reason for shunt failure is different across the three different types of VPS.

• To determine which organisms and their resistance/sensitivities subsequently infect these three alternative VPS.

• To determine whether antibiotic or silver-impregnated VPS reduce infection following the first (non-infected) clean VPS revision for mechanical failure compared to standard VPS.

• To assess the impact of VPS infection on the patient in terms of quality of life.

• To assess the cost-effectiveness of antibiotic-impregnated and silver-impregnated VPS compared to standard VPS.

## Methods/design

### Design overview

**Figure 1:**
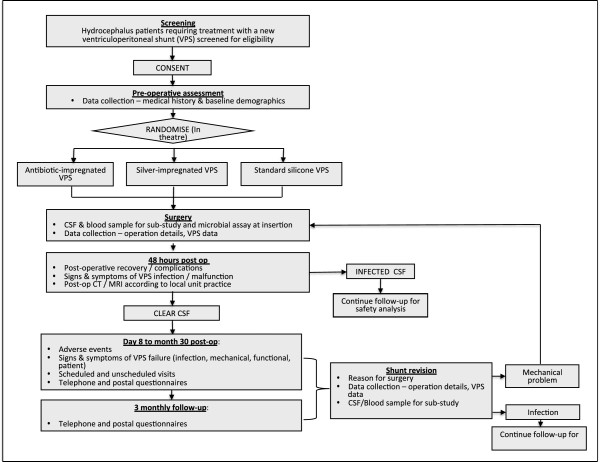
Flow diagram for trial.

The BASICS trial is a national three-arm, multi-centre, phase III RCT comparing antibiotic-impregnated, silver-impregnated, and standard VPS in patients with hydrocephalus undergoing insertion of their first permanent shunt. The design of the trial is presented in Figure 1.

### Research setting

The trial will be conducted across 17 paediatric and adult regional neurosurgery units in the UK and Ireland. A feasibility survey completed by 14 centres provided information on the number of eligible hydrocephalus patients and confirmed that the sample size is achievable.

### Funding and ethics approval

The BASICS trial is funded by a £2.04 m grant from the National Institute of Health Research Health Technology Assessment (NIHR-HTA) programme. CLM is the chief investigator and MDJ is the co-chief investigator. The study protocol, patient information sheets, and consent forms have received ethical approval from the North West Greater Manchester Research Ethics Committee (reference: 12/NW/0790).

### Study population

The trial will be open to all patients (children and adults) with hydrocephalus requiring treatment with a first permanent VPS who meet the eligibility criteria.

### Inclusion criteria

• Hydrocephalus of any aetiology (including idiopathic intracranial hypertension) requiring first VPS.

• Failed primary endoscopic third ventriculostomy allowed.

• Previous indwelling EVD allowed.

• Previous indwelling ventricular access device (for example Ommaya or Rickham reservoir or similar) allowed.

### Exclusion criteria

• Evidence of CSF infection prior to surgery for first VPS.

• Previous indwelling VPS.

• Active and on-going CSF or peritoneal infection.

• Multiloculated hydrocephalus requiring multiple VPS or neuroendoscopy.

• Ventriculoatrial or ventriculopleural shunt planned.

• Allergy to antibiotics associated with the antibiotic shunt.

### Primary outcome measure

Time to VPS infection following insertion of first *de novo* VPS for hydrocephalus.

### Secondary outcomes

• Time to shunt failure of any cause.

• Reason for shunt failure (infection, mechanical failure, patient failure, or other).

• Types of bacterial shunt infection (organism type and antibiotic resistance).

• Time to shunt infection following first clean revision.

• Quality of life.

• Incremental cost per shunt failure averted.

• Incremental cost per quality-adjusted life-year (QALY) gained.

### Randomisation

Patients will be randomised to standard, antibiotic, or silver-impregnated VPS in a ratio of 1:1:1. These are all CE marked medical devices used in accordance with the manufacturer’s instructions for their intended purpose. Written informed consent and assent, where appropriate, will be obtained pre-operatively, but randomisation will not occur until the trial participant is in the theatre suite so that only patients demonstrating continuing eligibility will be randomised at this stage, avoiding the randomisation of ineligible patients as far as is practically possible. Theatre staff cannot be blinded to the type of shunt inserted, but once the shunt is in position there will be no visible indications of the shunt type used. The type of shunt inserted will not be disclosed outside the operating theatre. Training on non-disclosure of shunt type will be provided to all staff members present at surgery. The majority of shunt revisions and removals for infection happen as emergencies and are managed by the on-call team. The likelihood of the same surgeon who inserted the shunt being involved in the decision to remove the shunt is low given the work rotas of surgical staff. However, this will be tracked by logging the staff involved in surgery and in the decision to remove the shunt. This process is the same as that successfully implemented for the catheter infections in children (CATCH) trial (ISRCTN34884569) and will form part of the trial monitoring report presented to the trial management group.

### Proposed sample size

Approximately 30% of new VPS will fail within the first 6 months of insertion due to shunt complications, which will include infection but also malfunction and mechanical failure, typically secondary to catheter or valve obstruction and peritoneal complications. When a VPS has failed for reasons other than infection then this prevents infection being observed. Infection is the event of interest with all other reasons for shunt failure being competing risks. The trial will compare antibiotic-impregnated versus standard VPS, and silver-impregnated versus standard VPS. A Bonferroni adjustment has been made to allow for the multiple comparisons and a statistical significance level of 0.025 will be used accordingly. The sample size calculation is based on Pintilie [24] assuming time to any type of event (of interest or competing risks) follows an exponential distribution. Using a 2-year accrual period with 6-month follow-up, once accrual has been completed and a competing risks event rate of 30% across trial arms with the event rate of interest (infection) being 8% in the standard arm and 4% in the treated arms (hazard ratio of 0.49), then with 5% loss to follow-up we would require 989 patients to provide 80% power. The power varies according to changes in the event rate with a fixed total sample size of approximately 1,143. Allowing for 5% loss to follow-up a sample size of 1,200 participants allows a hazard ratio of 0.49 to be detected over a range of baseline event rates (0.05 to 0.1) with good statistical power. The value of 8% is supported elsewhere [17], in addition, infection rate surveillance over 10 years (1993 to 2003) at Great Ormond Street Hospital, London, UK, involving over 1,500 insertions has demonstrated that while there are fluctuations in monthly infection rates that overall the rate has remained remarkably stable around this level. Observed fluctuations in infection rates within a centre will be influenced by the size of the denominator. Reduction of infection is a priority and therefore it could be argued that much smaller differences than those the study is powered to detect are still important. However the investigators believe that a strong effect is required to inform clinical practice and establish a first-line treatment policy across the National Health Service (NHS). This is in part due to the large differences in cost between the VPS; we need to warrant expenditure on the type of VPS as opposed to other infection control activities.

### Statistical analysis

The primary analysis will be by intention-to-treat principle as far as is practically possible and a full statistical analysis plan will be developed prior to comparative analysis. Results will be presented throughout using 97.5% confidence intervals and a 2.5% level of statistical significance. Analysis of the primary outcome will be by cumulative incidence. The Fine and Gray [25] regression method will be used to directly model the cumulative incidence function. In addition two semi-parametric models described in Scheike and Zhang [26] will be used to consider time-varying effects. Time-to-event outcomes where competing risks is not an issue will be analysed using Kaplan–Meir curves, log-rank tests, and Cox proportional hazard models. Assumptions of proportional hazards will be investigated. Categorical outcomes will be analysed using Chi-squared test. An interim analysis will be conducted halfway through the trial after approximately 50% of the total events have been observed using Haybittle [27].

### Heath economic analysis

The health economic analysis will adopt the perspective of the NHS in the UK and personal social services. Costs will include those of the catheters, duration of intensive care stay and hospital admission, antibiotic treatment, and contact with health professionals and social services. Resource use will be based on entries made in designated sections of patients’ case report forms which will include questions on use of personal social services [28], complemented with hospital.

Hospital Episode Statistics (HES) data will be sourced from the NHS Information Centre. Unit cost data will be obtained from routine hospital data (NHS reference costs) [29] and other standard sources [30,31]. Given that the economic question under consideration is one of technical efficiency (that is, the aim to maximise health outcomes given the resource inputs), we shall approach this as a cost-effectiveness analysis, based on the incremental cost per shunt failure averted. Secondary economic outcomes will include: 1) incremental cost per shunt infection avoided; and 2) incremental cost per QALY gained, estimated by administering the EuroQol (Rotterdam, Netherlands) EQ-5D-3 L, EQ-5D-Y (youth version), or Health Utilities Index Mark 2 (HUI2; Dundas, ON, Canada) to patients (or their parents, according to age) at each follow-up visit. Costs and benefits occurring after the first year will be discounted at 3.5% per annum. Total costs will be combined with the measures of health outcome to calculate the incremental cost-effectiveness (utility) ratios of each technology. Where appropriate, missing resource use or health outcome data will be imputed. The number of QALYs experienced by each patient will be calculated as the area under the curve, using the trapezoidal rule, and corrected for baseline utility score. Non-parametric bootstrapped 95% confidence intervals will be estimated (10,000 replicates). We will also employ parametric approaches for analysing cost and QALY data that assume normal distributions given the large samples where the near-normality of sample means is approximated. Should the data indicate otherwise, we will develop a generalised linear model to deal with problems such as skewness. Stratified cost-effectiveness analyses will be conducted on important, pre-specified patient subgroups (including neonates, patients under 1 year old, and patients with a previous EVD). Cost-utility estimates will be compared with the £20,000 to £30,000 per QALY threshold of cost-effectiveness, specified by National Institute for Health and Care Excellence (NICE), and a range of one-way sensitivity analyses will be conducted to assess the robustness of the analysis. Multivariate sensitivity analyses will be applied where interaction effects are suspected, for instance in the assessment of the combined effect of development of resistant organisms and the costs associated with their management. The joint uncertainty in costs and benefits will be considered through the application of bootstrapping and the estimation of cost-effectiveness acceptability curves [32].

### Translational research studies

The study has ethical approval and funding to obtain an additional 5 mL of blood and CSF gifted by patients at VPS insertion and revision. These samples will be stored until trial completion and then used for research studies to characterise the blood and CSF patterns of host response during confirmed shunt infection (via molecular methods including proteomics and transcriptomics), with an aim to use these patterns to strengthen the diagnosis of shunt infections and improve pathogen detection.

### Dissemination of results

The communication and dissemination strategy will actively involve participating centres, their staff and service users, professional bodies involved (Society of British Neurological Surgeons, SBNS), and relevant charitable organisations (spina bifida, hydrocephalus, information, networking, equality, Shine). Communication and dissemination of results will be assisted by members of the study team, including using social networking sites. Findings of the trial will also be presented at national and international meetings of relevant professional bodies and research groups. The results of the trial will be published in peer-reviewed journals.

If the trial favours a particular impregnated catheter, we will initiate a forum for discussion with other specialties such as neurologists, microbiologists, and infection control specialists and industry to discuss the implications for practice for research into impregnated catheters for other patient groups requiring implants in the central nervous system, such as baclofen pumps and catheters, spinal cord stimulators, and deep brain stimulation.

## Discussion

This protocol describes the design of a RCT to evaluate the clinical effectiveness and cost-effectiveness of antibiotic- and silver-impregnated catheters to reduce VPS infection. This is the first RCT to compare these catheters with conventional non-impregnated silicone catheters. This study will generate one of the largest CSF and blood biobanks in a cohort of hydrocephalus patients.

## Trial status

The trial opened for recruitment in June 2013 (http://www.basicsstudy.org.uk). Up to 30 November 2013, eight centres have opened and 49 patients have been randomised.

## Abbreviations

BASICS trial: British antibiotic and silver-impregnated catheters for ventriculoperitoneal shunts multi-centre randomised controlled trial; CATCH: Catheter infections in children; CE: Conformité Européenne; CI: Confidence interval; CNS: Central nervous system; CSF: Cerebrospinal fluid; EVD: External ventricular drain; HES: Hospital episode statistics; HR: Hazard ratio; IQ: Intelligence quotient; NHS: National Health Service; NICE: National Institute for Health and Care Excellence; NIHR-HTA: National Institute of Health Research Health Technology Assessment; QALY: Quality-adjusted life-year; RCT: Randomised controlled trial; RR: Relative risk; SBNS: Society of British Neurological Surgeons; Shine: Spina bifida, hydrocephalus, information, networking, equality; VPS: Ventriculoperitoneal shunt.

## Competing interests

The authors report no competing interests.

## Authors’ contributions

CLM and MDJ conceived the trial question and have been involved in all stages of the study design, and together with CG, MB, HH, JH, DH, and TS participated in writing the protocol, submission to the funding body, and application to the ethics committee. CG and MB are the trial statisticians. HH is a senior trials manager who coordinated submission. JH has defined the microbiology input for the trial. DH is the health economist and designed the health economic analysis. TS and MJG developed the basic science studies for shunt infection and host response. All authors read and approved the final manuscript.
